# Myiasis associated with penile carcinoma: a new trend in developing countries?

**DOI:** 10.1590/S1677-5538.IBJU.2016.0084

**Published:** 2017

**Authors:** Leandro Koifman, Rodrigo Barros, Lucas Schulze, Antonio Augusto Ornellas, Luciano A. Favorito

**Affiliations:** 1 Hospital Municipal Aguiar Souza, RJ, Brasil;; 2 Serviço de Urologia, Hospital Mário Kröeff, RJ, Brasil;; 3 Departamento de Urologia, Instituto Nacional de Câncer, RJ, Brasil;; 4 Unidade de Pesquisa Urogenital - Universidade do Estado do Rio de Janeiro, RJ, Brasil

**Keywords:** Penile Neoplasms, Myiasis, Lymph Node Excision

## Abstract

**Objectives:**

The aim of this study is to report an unusual form of penile cancer presentation associated with myiasis infestation, treatment options and outcomes.

**Materials and Methods:**

We studied 10 patients with suspected malignant neoplasm of the penis associated with genital myiasis infestation. Diagnostic assessment was conducted through clinical history, physical examination, penile biopsy, larvae identification and computerized tomography scan of the chest, abdomen and pelvis. Clinical and pathological staging was done according to 2002 TNM classification system. Radical inguinal lymphadenectomy was conducted according to the primary penile tumor pathology and clinical lymph nodes status.

**Results:**

Patients age ranged from 41 to 77 years (mean=62.4). All patients presented squamous cell carcinoma of the penis in association with myiasis infestation caused by *Psychoda albipennis*. Tumor size ranged from 4cm to 12cm (mean=5.3). Circumcision was conducted in 1 (10%) patient, while penile partial penectomy was performed in 5 (50%). Total penectomy was conducted in 2 (20%) patients, while emasculation was the treatment option for 2 (20%). All patients underwent radical inguinal lymphadenectomy. Prophylactic lymphadenectomy was performed on 3 (30%) patients, therapeutic on 5 (50%), and palliative lymphadenectomy on 2 (20%) patients. Time elapsed from primary tumor treatment to radical inguinal lymphadenectomy was 2 to 6 weeks. The mean follow-up was 34.3 months.

**Conclusion:**

The occurrence of myiasis in the genitalia is more common in patients with precarious hygienic practices and low socio-economic level. The treatment option varied according to the primary tumor presentation and clinical lymph node status.

## INTRODUCTION

Penile cancer is a rare neoplasm which treatment causes devastating effects on the patient’s physical and mental health. The low incidence of this disease in developed countries in contrast to the high incidence in developing countries clearly indicates the disease’s association with local economic conditions (1-4). Although in the United States the incidence rate of penile cancer accounts for 0.2 cases per 100.000 inhabitants, in Brazil this rate ranges from 2.9 to 6.8 per 100.000 inhabitants. As a result, Brazil is ranked as one of the countries with the highest incidence of this neoplasia in the World (4, 5).

There are few epidemiological studies conducted in patients with penile carcinoma (4, 5). In a recent series, the authors established an epidemiological profile in which patients had a very low socio-economic status with low education, tending to delay seeking medical help, and therefore the diagnosis of the disease is frequently performed in advanced stages (5).

Myiasis is defined as a disease caused by the infestation of larvae or maggots of numerous flies species that grow inside a host, while feeding on the host’s tissue. Such flies are usually attracted to open wounds and urine or feces-soaked fur (6). The incidence of myiasis is more commonly observed in rural areas as well as socioeconomically underdeveloped regions with precarious hygiene conditions (6, 7). The occurrence of myiasis in the genitalia is rare (7), especially when linked to penile cancer (8).

The aim of this study is to report an unusual form of penile cancer presentation associated with myiasis infestation, treatment options, and outcomes.

## MATERIALS AND METHODS

**Figure 1:**
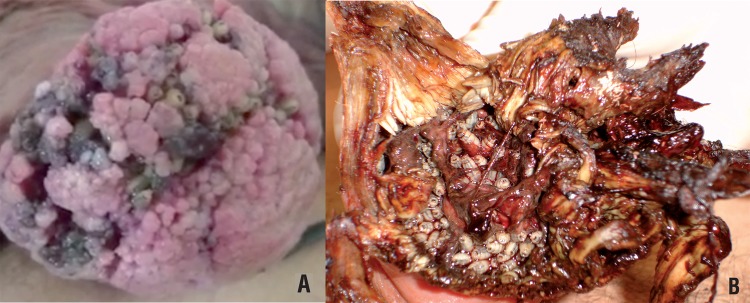
Penile cancer associated to myiasis. A) 44 year-old patient with extensive lesion in penile shaft with secondary infection associated with myiasis infestation.

**Figure 2:**
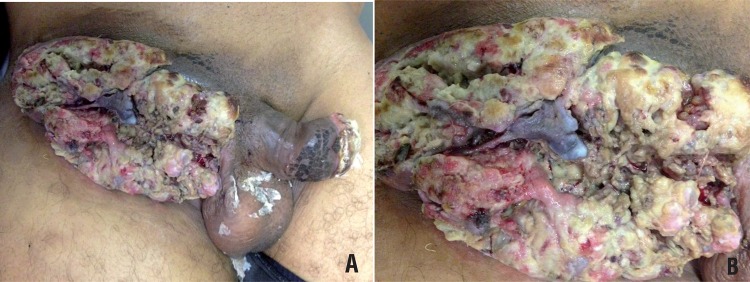
Penile cancer associated to inguinal myiasis. A) We can observe a 60 year-old patient with extensive inguinal metastasis due to penile cancer. B) In high magnification we can observe the inguinal metastasis infested by myiasis.

Between January 2003 and July 2014, 10 patients with suspected malignant neoplasm of the penis associated with genital myiasis infestation were admitted in our emergency room facility (Figures 1 and 2). Primary diagnostic assessment was conducted through clinical history and physical examination. All patients presented genital tissue infection and were primarily treated with a combination of venous antibiotics (Ciprofloxacin and Clindamycin), started on hospital admission, totaling 21 days, and oral single dose of Ivermectin (150mcg/Kg) for parasitic infection. All patients underwent larvae manual removal and biopsy of the primary lesion for diagnostic confirmation under anesthesia. Larvae taken from the primary penile tumor were sent to the laboratory for classification.

(Added with permission of Int Braz J Urol).

Epidemiological variables evaluated in this study were: age, ethnicity, educational level, smoking, presence of phimosis, practice of circumcision and clinical history of sexually transmitted diseases.

Patients were clinically evaluated for the presence of inguinal and visceral metastases by physical examination of the inguinal region and computerized tomography scan of the chest, abdomen and pelvis. Clinical and pathological staging was done according to 2002 TNM classification system. Clinical characteristics of the primary lesion as well as the clinical TNM classification are described in Table-1.

**Table 1:** Clinical characteristics of primary lesion, patient’s age, anatomical area of myiasis infestation, primary tumor location, and clinical 2002 TNM classification.

Age	Tumor Location	Area of Myiasis	Clinical Characteristics Primary Lesion	TNM
41	Glans and Penile Shaft	Glans	8 cm exophytic lesion with gross inflammatory and infectious signs	CT3N1M0
55	Glans, Penile Shaft and Scrotum	Penile shaft	12 cm genital lesion involving glans and penile shaft with extensive ulcerated area with gross inflammatory and infectious signs	CT4N2M0
65	Glans and Penile Shaft	Glans and Penile Shaft	7 cm ulcerated lesion with gross inflammatory and infectious signs	CT3N1M0
60	Glans, Penile Shaft and Scrotum	Glans, Penile Shaft, Scrotum and Inguinal Area	7 cm exophytic lesion with gross inflammatory and infectious signs	CT4N3M0
62	Glans	Glans and Inguinal Area	4 cm exophytic lesion with gross inflammatory and infectious signs	CT3N3M0
67	Prepuce and Glans	Prepuce and Glans	5 cm ulcerated lesion with gross inflammatory and infectious signs	CT2N0M0
72	Prepuce and Glans	Prepuce and Glans	5 cm exophytic lesion with gross inflammatory and infectious signs	CT2N0M0
70	Prepuce and Glans	Prepuce and Glans	4,5 cm exophytic lesion with gross inflammatory and infectious signs	CT2N0M0
55	Prepuce and Glans	Prepuce and Glans	4 cm exophytic lesion with gross inflammatory and infectious signs	CT2N2M0
77	Prepuce and Glans	Prepuce and Glans	5 cm exophytic lesion with gross inflammatory and infectious signs	CT2N2M0

Pathological material was reviewed and all tumors were histologically classified based on Broder’s system. Only two pathologists were responsible for reviewing the primary penile lesions and lymphadenectomy specimens. The pathological variables studied were the histological type, grade, size of the lesion, corpus spongiosum and/or corpora cavernosa infiltration, urethral infiltration, and lymphovascular involvement.

The type of treatment for the primary tumor of each patient was included in this study. All patients who were indicated for adjunctive treatment of inguinal lymphatic basins underwent radical bilateral inguinal lymphadenectomy. We considered lymphadenectomy to be prophylactic when performed on patients with clinically negative lymph nodes and high risk of inguinal dissemination (PT2 and/or lymphovascular invasion and/or Broders histological classification greater than or equal to II). We considered it to be therapeutic when performed on patients with clinically positive inguinal lymph nodes. Finally, we considered it to be palliative for patients with large ulcerated tumor masses and/or masses fixed in the inguinal region. The time elapsed from the primary tumor treatment and radical inguinal lymphadenectomy was evaluated.

All patients were evaluated prospectively and provided informed consent to participate in the study. Our institutional review board also approved the study. The mean follow-up was 34.3 months.

## RESULTS

Patient’s age ranged from 41 to 77 years (mean=62.4). Of the 10 patients evaluated, 9 (90%) were white and 1 (10%) black. The level of education varied from illiterate in 8 (80%) patients, to high-school graduate in 2 (20%) patients.

Among the evaluated patients, 8 (80%) were homeless while 2 (20%) lived in supported geriatric home. In this series, all patients were tobacco smokers and only 1 (10%) had been circumcised in adolescence. The remaining 9 (90%) patients presented phimosis. Only 2 (20%) patients reported history of sexually transmitted diseases, presented as urethritis. The remaining 8 (80%) patients were not able to report or denied sexually transmitted diseases.

In relation to the pathological variables studied, all patients presented squamous cell carcinoma of the penis. The lesion size ranged from 4cm to 12cm (mean=5.3). The treatment option for the patients varied according to the presentation of the primary tumor (Table-1). Circumcision with partial amputation of the glans was conducted in 1 (10%) patient while partial penectomy was performed in 5 (50%) patients. Total penectomy was conducted in 2 (20%) patients while emasculation was the treatment option for 2 (20%) patients with extensive involvement of the penile shaft and scrotum. All patients underwent bilateral inguinal radical lymphadenectomy to complement the treatment of the primary lesion. Prophylactic lymphadenectomy was carried out on 3 (30%) patients while therapeutic lymphadenectomy was conducted on 5 (50%) patients. The remaining 2 (20%) patients were submitted to palliative lymphadenectomy. Chest, abdomen, and pelvis computerized tomography done systematically to stage all cases revealed no visceral metastasis or pelvic lymphadenopathy suggesting tumor spread. Time elapsed from primary tumor treatment to inguinal lymphadenectomy was 2-6 weeks in 5 (50%) patients while in 5 (50%) remaining patients, both procedures were performed simultaneously.

The larvae collected from the penile tumors and sent to the laboratory were classified as *Psychoda albipennis*, a species from the family *Psychodidae* and gender *Psychoda*. Treatment for parasitic infestation was effective with no detected larvae in surgical specimens.

Pathological characteristics of the primary penile tumor and lymph node are represented in Table-2.

**Table 2:** The table shows the pathological features and surgical stage according to 2002 TNM classification of the 10 patients with penile cancer associated to myiasys.

		TUMOR STAGE		

Pathologycal features	pT1 (%)	pT2 (%)	pT3 (%)	pT4 (%)
G1	0	0	0	0
G2	0	4 (40)	2(20)	2 (20)
G3	0	1 (10)	1 (10)	1 (10)
Lymph Invasion +	0	4 (40)	3 (30)	2 (20)
Lymph Invasion -	0	1(10)	0	0
pN0	0	3 (30)	2 (20)	0
pN1	0	0	0	0
pN2	0	2 (20)	1 (10)	0
pN3	0	0	0	2 (20)

## DISCUSSION

Urogenital myiasis is an extremely rare condition seen in immunocompromised individuals, elderly, and persons with poor personal hygiene. It commonly occurs in tropical, subtropical countries, and areas with warm climate (9-11).

The most common form of myiasis in men takes place in the skin, where the species Dermatobia hominis is mostly observed. The severity of the condition depends on the location and on the degree of tissue destruction (9-11). *Psychoda albipennis* is an insect species that causes urogenital myiasis in humans. Adult forms of this species belongs to the *Psychodidae* subfamily, and lives especially in humid toilets and domestic bathrooms (10). Flies are attracted to malodor and suppurative lesions where they lay their eggs and develop into larvae. The pathogenicity results from inflammation and toxins secreted by the larvae. The larvae are photophobic, penetrating deep into the tissues with the help of sharp mouth hooks. Genitourinary infestation usually presents as pain and pruritus at the site (8-11). Transmission occurs through the accidental deposit of eggs on oral or genitourinary openings, or by swallowing eggs or larvae that are present on food (9).

The myiasis larvae can develop in two clinical cases: obligate parasites, which thrive on living tissues, and facultative parasites, which attack necrotic tissues and wounds. The larvae generally found in necrotic lesions (cavitary myiasis) are from the genera: *Sarcophaga, Lucilia, Calliphora and Musca*. Genital myiasis can cause unique ulcerated lesions that are often confused with sexually transmitted diseases (12).

The male genital infestation is rare, since the area is usually protected by clothes, and is, therefore less accessible to insect’s contact (13-15). In the present series 8 (80%) patients were homeless while 2 (20%) patients lived in support geriatric home. Lyra (16) described a case of a 20-year-old military soldier with furuncular myiasis on penile glans. Two weeks earlier, he had returned from a military mission in a rural area with poor hygiene conditions. The precarious hygiene conditions of such patients justified an adequate environment for myiasis infestation, especially when penile cancer is present, with open wound areas and necrotic tissues.

The etiology of penile cancer has not been fully elucidated. However, its incidence varies according to the practice of circumcision, personal hygiene, presence of phimosis, human papilloma virus infection and tobacco use (4, 5). Despite the level of education varying from illiterate to high-school graduate in the present series, all patients presented with deplorable hygiene conditions at their hospital admission. In this series, all patients were tobacco smokers and only 1 (10%) had been circumcised in adolescence. The remaining 9 (90%) patients presented phimosis.

The 2002 TNM classification for penile cancer, has been criticized by several authors (17-19). Because it is essentially a pathological assessment, it is virtually impossible to clinically determine the precise level of tumor invasion and the real lymph node status. In the study conducted by Petralia (20), physical examination was able to properly stage the primary tumor in only 8 of 13 patients (61.5%), with overstaging in 2 (15.4%) and understaging in the other 3 (23.1%) patients. Likewise, de Kerviler (21) only obtained a correct clinical staging of penile lesions in 66.6% of patients in their series. In another study conducted by Koifman (5) the authors observed clinical staging accuracy of the primary tumor in 75.2% of 230 patients evaluated.

In the present series we observed clinical staging accuracy of the primary tumor in 50% of cases. When stratifying patients according to the primary tumor, understaging was observed in 25% of patients with T2 and 33.3% of patients with T3, while overstaging took place in 20%, 33.3% and 50%, respectively for T2, T3, and T4 tumors. Misinterpretation of the degree of tumor infiltration of the primary lesion on physical examination could be attributed to local edema, infectious processes that arise at the tumor site and mass effect caused by the presence of the larvae.

The central mechanism responsible for tissue repair after injury is inflammation. Malignant neoplasms use deficiencies in the repair mechanisms to maintain cell growth and proliferation. This double face of inflammation process intended to ensure tissue repair, may undergo changes in their orientation, contributing to the growth and development of neoplasia. The disordered production of inflammation factors by the tumor leads to the blockage of natural apoptosis process (22, 23). In a recent study conducted by Koifman (24) the authors demonstrate through proteomic analysis, the absence of human complement C3 in samples of patients with squamous cell carcinoma of the penis. A possible explanation for these findings lies on the theory that patients with malignancies have a poorer immune response. It is possible that the presence of myiasis in association with penile carcinoma intensify local inflammatory process, creating an ideal environment for tumor proliferation.

The association between myiasis and penile cancer is extremely rare with only 2 reports published in the international literature. Tavares (8) described the first case in the literature. Singh (25) published a case of myasis associated with carcinoma in situ of penile glans.

In the present study, it was possible to observe the process of misinformation among individuals with precarious hygiene habits, leading to the exacerbation of a condition that could have been tackled with a less aggressive treatment, in an initial phase, with proper earlier diagnoses. The association between myiasis and penile carcinoma reinforce the need to implement new awareness campaigns on penile cancer in developing countries.

The occurrence of myiasis in the genitalia area is rare, especially when associated with penile cancer. This condition mainly affects patients with a very low socioeconomic status, characterized by poor hygienic habits. Poorer patients with less education tend to delay longer in seeking medical care and therefore the diagnosis of the disease is frequently performed in advanced stages. To our knowledge this study represents the first series of patients diagnosed with genital myiasis in association with penile carcinoma.

## CONCLUSIONS

The occurrence of myiasis in the genitalia is more common in patients with precarious hygienic practices and low socio-economic level. The treatment option varied according to the primary tumor presentation and clinical lymph node status.

## ARTICLE INFO

Int Braz J Urol. 2017; 43: 73-9
